# Expansion of *C9ORF72* in amyotrophic lateral sclerosis correlates with brain-computer interface performance

**DOI:** 10.1038/s41598-017-08857-3

**Published:** 2017-08-21

**Authors:** Andrew Geronimo, Kathryn E. Sheldon, James R. Broach, Zachary Simmons, Steven J. Schiff

**Affiliations:** 1 Penn State College of Medicine, Department of Neurosurgery, Hershey, PA 17033 USA; 2 Penn State University, Center for Neural Engineering, University Park, PA 16802 USA; 3 Penn State College of Medicine, Department of Biochemistry and Molecular Biology, Hershey, PA 17033 USA; 4 Penn State College of Medicine, Departments of Neurology and Humanities, Hershey, PA 17033 USA; 5 The Pennsylvania State University, Departments of Engineering Science and Mechanics, and Physics, University Park, PA 16802 USA

## Abstract

Abnormal expansion of hexanucleotide GGGGCC (G_4_C_2_) in the *C9ORF72* gene has been associated with multiple neurodegenerative disorders, with particularly high prevalence in amyotrophic lateral sclerosis (ALS) and frontotemporal dementia (FTD). Repeat expansions of this type have been associated with altered pathology, symptom rate and severity, as well as psychological changes. In this study, we enrolled twenty-five patients with ALS and fifteen neurologically healthy controls in a P300 brain-computer interface (BCI) training procedure. Four of the patients were found to possess an expanded allele, which was associated with a reduction in the quality of evoked potentials that led to reduced performance on the BCI task. Our findings warrant further exploration of the relationship between brain function and G_4_C_2_ repeat length. Such a relationship suggests that personalized assessment of suitability of BCI as a communication device in patients with ALS may be feasible.

## Introduction

The repeat hexanucleotide GGGGCC (G_4_C_2_) expansion in the gene *C9ORF72* is the most common identified genetic link between amyotrophic lateral sclerosis (ALS) and frontotemporal dementia (FTD). With an upper limit of normal defined as thirty repeats^1, 2^, abnormal expansion of this sequence is prevalent in 50–72% of patients with familial ALS^3^, and roughly 7% of sporadic cases^4^.

ALS patients possessing the repeat expansion exhibit earlier onset, more rapid progression, and earlier death^4–10^. MRI studies point to structural changes common among repeat carriers that extend beyond the degeneration of the cortico-motor pathways typically involved in ALS^7, 11, 12^. Transcranial magnetic stimulation studies have identified a reduction in cortical inhibition attributable to this expansion found in ALS patients^13, 14^. In an electroencephalographic (EEG) study of seven ALS/FTD patients with the repeat expansion, two showed generalized slowing of the background activity, while another two showed intermittent abnormal temporal delta-theta activity^15^. Another study described a bilateral slowing of frontotemporal theta in three individuals with the expansion^16^.

Brain-computer interfaces (BCI), specifically the P300 speller^17^, enable communication without overt movement. The visual P300 spelling task relies on a user’s focus on an intended target while observing a larger group of randomly flashing letters. Reaction to flashing of the target, although producing no overt disturbance, generates a P300 evoked potential over centro-parietal electrode sites, which the computer can interpret as a selection. In the past few years, these devices have been used successfully as assistive communication tools for those with ALS^18–20^, but declines in performance with severe disease progression have also been documented^21–23^. The effect of cognitive decline on the performance of these devices has recently been shown to be a major predictor of BCI utility in this population^24^. The question of whether genotype, particularly in this case as it applies to expansion of *C9ORF72*, produces measurable changes in the performance of an EEG-based BCI task is unexplored. Our hypothesis is that the BCI task in this study - evocation of the P300 potential and related visually evoked potentials (VEPs) - is performance-dependent on the absence of *C9ORF72* expansion. In this paper, we describe a relationship between this repeat expansion and EEG modulation for the purpose of brain-computer interaction.

## Results

**Figure 1:**
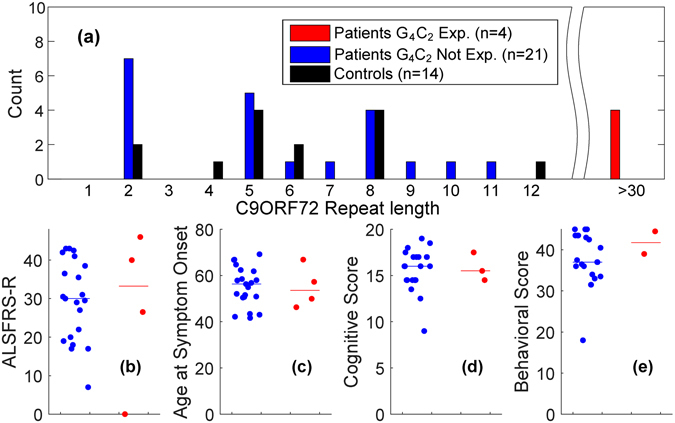
(**a**) Histogram of *C9ORF72* allele lengths. The presence of a repeat expansion among ALS patients (horizontal line indicates group median) does not significantly associate with (**b**) ALSFRS-R score, (**c**) age at symptom onset, (**d**) cognitive impairment, or (**e**) behavioral impairment.

Twenty-five patients were enrolled along with 15 neurologically-healthy control participants who were age and gender matched (Table 1). One patient did not complete the ALS Cognitive Behavioral Screen (ALS-CBS) because of severe communication difficulties, and another did not complete the behavioral portion of the screen because of the lack of an available caregiver. All patients and 14/15 controls provided samples for assessment of the *C9ORF72* repeat expansion. Therefore, 25 patients and 14 control participants were included in the analysis. All control participants possessed hexanucleotide repeat lengths of twelve or fewer (Fig. 1a). Four out of 25 patients (16%) carried a repeat expansion. The presence of a hexanucleotide repeat expansion was not found to be associated with ALS Functional Rating Scale (ALSFRS-R) score, age at symptom onset, nor with cognitive or behavioral scores on the ALS-CBS (Fig. 1b–e).

**Table 1:** Patient and control participant demographics.

	Patients (n = 25)	Controls (n = 14)	p-val
Age, years	58 (45.5–74)	55.3 (45–73.5)	0.292
Education, years	14 (11.5–24)	18 (12–24)	0.005
Gender, % male	68	57	0.515

Statistics are given as median and range (min-max) and p-values are the result of Wilcoxon rank sum testing between control and patient samples.

**Figure 2:**
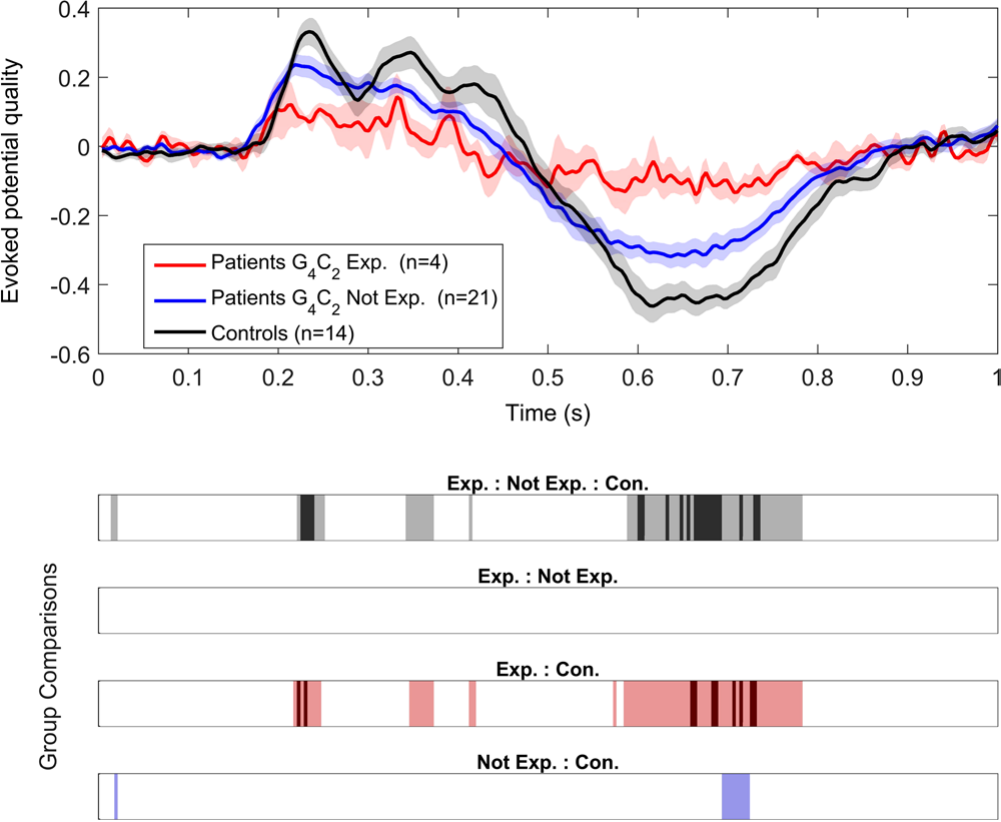
VEP quality is decreased in ALS, particularly in the presence of *C9ORF72* expansion. Top: Mean quality of the evoked potential (±SEM) for controls and patients, with and without the expansion. Bottom: Windows where there exist significant differences in VEP quality between the three groups as well as three post-hoc pairwise comparisons. These differences are shown a the *α* = 0.05 level (light colors) as well as after FDR correction (dark colors).

A VEP quality metric, independent of the BCI classifier, was calculated to represent the discriminability of target vs. non-target EEG responses in the channels Fz, Cz, and Pz. Morphology of VEP quality differed between groups (Fig. 2). Control participants displayed greater magnitude quality responses compared to patients in both early (220–250 ms, 300–370 ms) and late (580–780 ms) evoked potentials. Differences can be seen in both of these periods between patients with and without the repeat expansion, although group-wide differences in median quality exist primarily between patients with the expansion and controls, most notably during the early and late portions of the VEP. The lower half of Fig. 2 shows the time windows in which the 3 group or pairwise comparisons generated a p-value of less than 0.05 (light shading), while the darker shading represent windows retaining significance after False Discovery Rate (FDR) correction (p < 0.0076).

**Figure 3:**
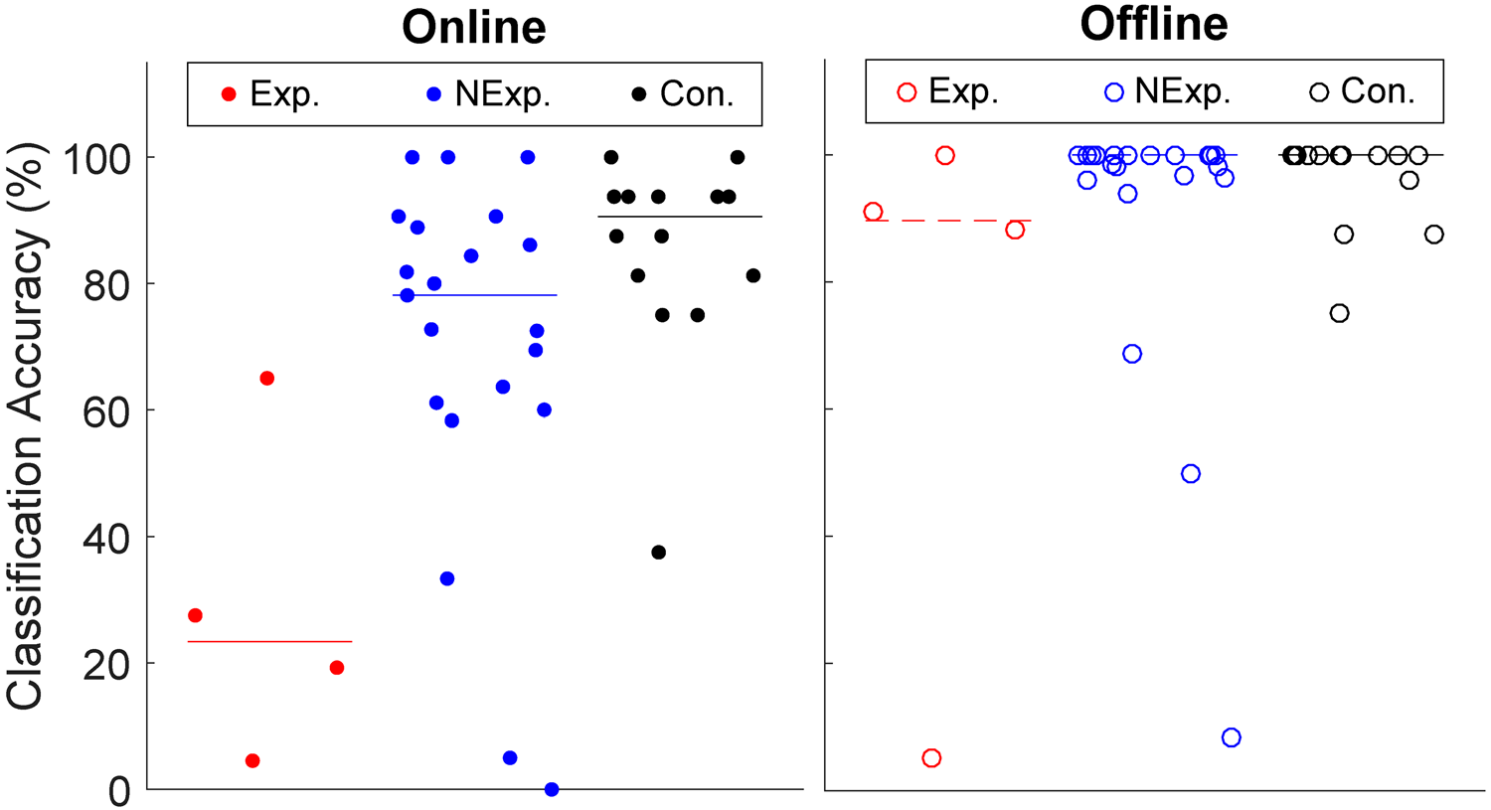
BCI accuracy for all study participants, with group medians represented by a horizontal line. Left: Median online accuracy for patients with the expansion was significantly less than controls (p = 0.005). Right: When analyzed offline, reduction in median accuracy in those with the expansion did not reach significance.

Online accuracies across groups varied significantly (Fig. 3). The median online P300 spelling accuracy was 23.4% for the patients with the *C9ORF72* expansion, 78.1% for patients without the expansion, and 90.6% for controls. The mean online accuracy ranks for each group were found to be significantly different (*χ* ^2^ = 10.67, p = 0.005). Post-hoc analysis showed that four patients with the expansion had lower mean rank than the control group (p = 0.005) and there was a non-significant trend to the same effect with the patients without the expansion (p = 0.095). The median offline P300 spelling accuracy was 89.7% for the patients with the *C9ORF72* expansion, 100.0% for patients without the expansion, and 100.0% for controls. The accuracies calculated offline were not found to differ significantly between the three groups (*χ* ^2^ = 3.43, p = 0.18).

## Discussion

Four of the ALS patients in this study possessed an expansion of the *C9ORF72* gene, which has been linked to the presence of electrophysiological brain abnormalities, including cortical hyperexcitability^13, 14^, background rhythm slowing^15^, and epileptic activity^16^. While there was both a reduction in VEP quality and online BCI performance in ALS patients as a group, those with the *C9ORF72* expansion demonstrated further reductions compared to those without it. This effect was not observed when comparing offline task accuracies across groups. This may be due to a classifier saturation effect; by the completion of the fourth session, patients with the expansion may have accumulated enough data to counteract the noisier signal and create a more generalizable classifier. The comparatively poor online accuracy for these patients may reflect a longer “learning period” for the classifier. In addition, offline accuracy from the control group was calculated with data from only two sessions, which may have also led to a relatively smaller difference in accuracies between patients and controls compared to online results.

The reduced discriminability of target vs. non-target EEG responses was observed most strongly in the early (220–250 ms) and late (580–780 ms) portions of the VEP. This was subclinical, in that those displaying the expansion were no different from patients without the expansion in regards to cognitive and behavioral function or symptom onset. We tried to control for other aspects of the disease which may better explain the observed differences. While we did account for age, time since symptom onset, and education, we did not measure vision or oculomotor control, which has been shown to be a critical factor in P300 speller success for those with ALS^22^.

Our results raise the possibility of a relationship between an expansion in the *C9ORF72* gene and brain function in patients with ALS. Previous work by our group showed that intact cognition, as measured by the ALS-CBS, was the disease characteristic most strongly predictive of P300-BCI performance^24^. With this present finding, differences in evoked potential production and BCI performance may be at least partially explained by physiological brain changes that are dependent on G_4_C_2_ repeat number, independent of ALS-CBS.

Although the study was performed prospectively, there were a few limitations to the procedure which limit the generalizability of these findings. The patients possessing gene expansion did not have a higher incidence of FTD symptoms. Since the presence of cognitive impairment with *C9ORF72* mutation is several-fold greater than those without (40–50% vs. 8–9%, respectively)^3^, we expected our group of four carriers to present with higher incidence of cognitive and/or behavioral impairments of FTD. We are aware, however, that our method for performing neuropsychological screening was far from comprehensive. This limitation and the small sample size may contribute to this missed association which has wide pathological and clinical backing. Better quantification of repeat expansions are also needed, in order to determine whether differences exist between individuals with intermediate repeat lengths (20–100), and large repeats (100–1000+)^25^. Although we are confident of our analysis leading to identification of patients harboring expansions, we did not have the ability to perform Southern Blotting for determination of precise expansion lengths.

The presence of hexanucleotide repeat expansions that affect BCI usage would have direct applicability for assessment of device utility, and thus could impact effectiveness of such devices in clinical use. Screening for repeat length in ALS patients might serve as one of several tools for personalized assessments of BCI suitability, or might guide the development of training programs for such devices. More broadly, this association raises the possibility of BCI as a diagnostic tool for early detection of non-motor manifestations of ALS.

## Methods

The study was approved by the Institutional Review Board of Penn State Hershey Medical Center. All methods were performed in accordance to the guidelines and regulations of The Pennsylvania State University. All participants provided informed consent and were at least 18 years of age. Those in the patient cohort possessed a diagnosis of definite, probable, probable laboratory-supported, or possible ALS by revised El Escorial research criteria^26^. Those with clinically significant dementia, as determined by the ALS clinic neurologist, were excluded. The control cohort consisted of neurologically healthy adults who were age and gender matched to the patient group. Age, gender, region of onset, time since symptom onset, education level, and ALS Functional Rating Scale - Revised (ALSFRS-R)^27^ were recorded from each patient. Cognitive and behavioral function were assessed via the ALS-Cognitive Behavioral Screen (ALS-CBS)^28^.

All subjects provided a saliva or blood sample for genetic analysis. Samples were initially analyzed by fluorescent fragment length polymerase chain reaction (FFL-PCR) to assay for the G_4_C_2_ repeat length^1^. For our FFL-PCR protocol, the largest callable repeat contained 14 G_4_C_2_ repeats. Samples determined to be heterozygotes by this method required no further analysis. Homozygotes underwent an additional assay using the Repeat Primed PCR (RP-PCR) method^1^, which was used to determine if the sample contained a true homozygous repeat number allele as opposed to a potentially pathogenic repeat that had been masked by FFL-PCR. Pathogenic repeats were identified qualitatively by the presence of a “stutter” pattern in the pherogram. In a fourteen-site blinded study, the mean sensitivity and specificity for detection of *C9ORF72* repeat expansion correctly from DNA was 95% and 98% respectively, when using this approach^29^. Fragment analyses were performed with GeneScan 500 ROX dye Size Standard on an ABI3130XL using Peak Scanner 2 software (Thermo Fisher Scientific).

Patients completed four sessions of EEG-BCI training over the course of 1–2 months, each with a half-hour training entailing 4–8 runs of a P300 spelling task. Control participants completed two sessions. Each run of the P300 spelling task consisted of copy spelling a 4 letter word, where each trial culminated in the selection of 1 letter after flashing each grid icon 20 times. A checkerboard-type speller with 32 targets^30^ was used to evoke the P300 signal from the user. Targets were highlighted for 187.5 ms, and the interstimulus interval was 62.5 ms.

During the recordings, EEG electrodes were affixed in an electrode cap at 19 locations in the 10–20 system, with ground at Fpz, and referenced to linked earlobes. Additionally, electrodes were placed on the forehead and lateral canthi for the purpose of artifact reduction. Electrode paste was used to mount the electrodes on the scalp, and impedances were generally kept below 10 kΩ. In this data set, we demonstrated previously that electrode impedance did not associate with BCI performance^24^. Signals were amplified and digitized with two g.USBamp amplifiers (g.tec GmbH). Data acquisition, signal processing, and feedback generation were performed by a customized program in BCI2000^31^. Ocular artifact reduction was automated though a regression procedure^32^, followed by rejection of epochs which exceeded an amplitude of ±75 *μ*V. Features forwarded to the classifier were the stimulus time-locked average EEG signals, downsampled to 20 Hz. Stepwise selection of regression coefficients was used on training data to generate a classifier for predicting intended targets online^30^. The classifier was generated using the *stepwisefit* function in MATLAB, and was updated after each feedback run.

Following all recordings, we utilized the Wilcoxon rank sum test to determine whether the presence of a repeat expansion was associated with cognitive or behavioral impairment, as well as age and ALSFRS-R score. Following this, the ‘quality’ of the BCI control signal was calculated. Signal quality acted as a classifier-independent measure of the robustness of the control signal. This metric was calculated for the BCI task as the standard distance between data belonging to the two classes of interest. As an example, *Q*_*Cz*_, the quality of the target-specific evoked potential in channel Cz, is the standard distance between target and non-target evoked potentials for the trials of that task,

1 $${Q}_{Cz}=\frac{{\mu }_{C{z}_{T}}-{\mu }_{C{z}_{N}}}{{\sigma }_{Cz}},$$

2 $${\sigma }_{Cz}=\sqrt{\frac{({n}_{T}-\mathrm{1)}\,{\sigma }_{C{z}_{T}}^{2}+({n}_{N}-\mathrm{1)}\,{\sigma }_{C{z}_{N}}^{2}}{{n}_{T}+{n}_{N}-2}},$$

where *Cz*_*T*_ represent the EEG responses in channel Cz to target stimuli and *Cz*_*N*_ represent the EEG responses to non-target stimuli, which occur at a ratio of 1:7. To compute the standard difference, the difference in means, *μ*, of these two groups are divided by their pooled standard deviation, *σ*.

Signal quality for the P300 task was averaged over channels Fz, Cz, and Pz. To determine whether there was a significant difference in the quality of the BCI control signals between groups, a series of Kruskal Wallis tests were performed at each EEG sample over one second following VEP extraction. Significant results were followed by post-hoc tests of pairwise comparisons using the Tukey-Kramer method, if necessary. To correct for multiple comparisons, the type-1 error rate threshold was modified using the False Discovery Rate method^33^. Finally, we reanalyzed all the EEG data offline using the same feature extraction and classification scheme to determine the offline accuracy for each individual. Data was segmented in a leave-one-trial-out manner for estimation of offline accuracy.
